# Labile iron accumulation augments T follicular helper cell differentiation

**DOI:** 10.1172/JCI159472

**Published:** 2022-05-02

**Authors:** Yogesh Scindia, Borna Mehrad, Laurence Morel

**Affiliations:** 1 Department of Pathology, Immunology and Laboratory Medicine and; 2 Department of Medicine, University of Florida, Gainesville, USA.

## Abstract

T follicular helper (Tfh) cells are a subset of CD4^+^ T cells that are essential in the pathogenesis of systemic lupus erythematosus (SLE). Notably, iron is required for activated CD4^+^ T lymphocytes to sustain high proliferation and metabolism. In this issue of the *JCI*, Gao et al. showed that CD4^+^ T cells from patients with SLE accumulated iron, augmenting their differentiation into Tfh cells and correlating with disease activity. Using human cells and murine models, the authors demonstrated that miR-21 was overexpressed in lupus T cells and inhibited 3-hydroxybutyrate dehydrogenase-2 (BDH2). The subsequent loss of BDH2 drove labile iron to accumulate in the cytoplasm and promoted TET enzyme activity, *BCL6* gene demethylation, and Tfh cell differentiation. This work identifies a role for iron in CD4^+^ T cell biology and the development of pathogenic effectors in SLE. We await future investigations that could determine whether modulating iron levels could regulate Tfh cells in human health and disease.

## Iron metabolism in the immune system

Iron is a trace mineral necessary for many physiological processes, including erythropoiesis, immune function, and host defense. It is also required for cellular activities, such as DNA replication and repair. In addition, iron acts as a cofactor in the synthesis of heme- and iron-sulfur–containing enzymes and in the tricarboxylic acid cycle, where it is required for optimal mitochondrial function and biogenesis (1). Iron levels are tightly regulated by cytokines and acute-phase proteins: during inflammatory states, macrophages and hepatocytes sequester iron, preventing access to invading microbes, and promoting iron-limited erythropoiesis and altered immune cell metabolism (2).

## Essential role of iron in CD4^+^ T cell–induced pathology

CD4^+^ T cells play an essential role in the pathogenesis of autoimmune diseases, such as systemic lupus erythematosus (SLE). CD4^+^ T cells from patients with SLE are highly proliferative and metabolically active, requiring energy and iron. Consistently, activated T cells upregulate the iron import protein, transferrin receptor-1 (TfR1; ref. 3). Furthermore, hematopoietic cell—specific deletion of the heavy chain of ferritin, the intracellular iron storage protein, results in reduced numbers of lymphocytes, without affecting other leukocyte quantities, highlighting the essential nature of iron regulation to T lymphocytes (4).

Iron metabolism is relevant to many T cell–mediated diseases: iron deprivation attenuates experimental autoimmune encephalomyelitis (EAE), a T cell–dependent autoimmune mouse model of multiple sclerosis. Conversely, increased iron levels in activated T cells can protect poly(RC)-binding protein-1 from caspase-mediated proteolysis, thus stabilizing GM-CSF mRNA and, ultimately, driving inflammation to worsen EAE outcomes (5). In particular, inhibiting the conversion of cytochrome *c*–Fe^3+^ to cytochrome *c*–Fe^2+^ reduced inflammation in models of T cell–mediated disease, such as intestinal inflammation, EAE, psoriasis, and T cell–driven asthma (6), indicating that the ferrous (Fe^2+^) form of iron is essential to T cell effector function. Collectively these studies highlight the importance of iron in sustaining T cell metabolism.

## Increased iron content in lupus CD4^+^ T cells

T cell iron metabolism in lupus has not been investigated in detail. Zhao et al. have previously shown that, as compared with cells from healthy controls, CD4^+^ T cells from patients with SLE contain more intracellular iron as well as miR-21, linking iron homeostasis to global DNA methylation status in CD4^+^ T cells (7). Mechanistically, miR-21 inhibits 3-hydroxybutyrate dehydrogenase-2 (BDH2), the dehydrogenase that mediates the formation of 2,5-dihydroxybenzoic acid (2,5-DHBA), a cytoplasmic iron-binding molecule. Reduced 2,5-DHBA levels result in the accumulation of labile Fe^2+^ in the cytoplasm, which enhances the activity of the ten-eleven (TET) enzyme and, thereby, promotes DNA demethylation and gene expression. In this issue of the *JCI*, Gao et al. took the observation one step further, linking CD4^+^ T cell iron content with pathogenic differentiation (8).

T follicular helper (Tfh) cells are a specialized subset of CD4^+^ T cells that reside in the B cell zones of secondary lymphoid organs and help germinal center B cells generate high-affinity class-switched antibodies. Tfh cells are expanded in patients with SLE, as well as mouse models of the disease, and correlate with disease activity (9). Tfh cells from murine lupus models depend on glycolysis for ATP production, which, when targeted, attenuates disease severity (10). Pharmacologic or genetic targeting of IL-21, a cytokine produced by Tfh cells that promotes plasma cell differentiation, is similarly beneficial in mouse models (11).

Gao et al. showed that circulating CD4^+^ T cells from patients with SLE, especially those with active disease, expressed increased ferritin and possessed higher iron content than T cells from healthy controls (8). This observation was not merely due to an increased activation level of the T cells of patients with SLE, because the increased intracellular iron occurred in all T cell subsets, including naive T cells. The CD4^+^ T cell Fe^2+^ content strongly correlated with the frequency of Tfh cells, and iron supplementation enhanced the in vitro differentiation of Tfh cells from T cells obtained from healthy controls, while iron chelation had the reverse effect, suggesting a causal relationship between increased T cell content and Tfh cell differentiation in SLE. CD4^+^ T cells from MRL/lpr lupus-prone mice also displayed higher iron content, which also supports a causal relationship. Moreover, treatment of the animals with a long-term high-iron diet increased T cell activation and inflammatory cytokine production, increased the frequency of Tfh and germinal center B cells, enhanced anti-dsDNA IgG production, and accelerated renal pathology.

**Figure 1:**
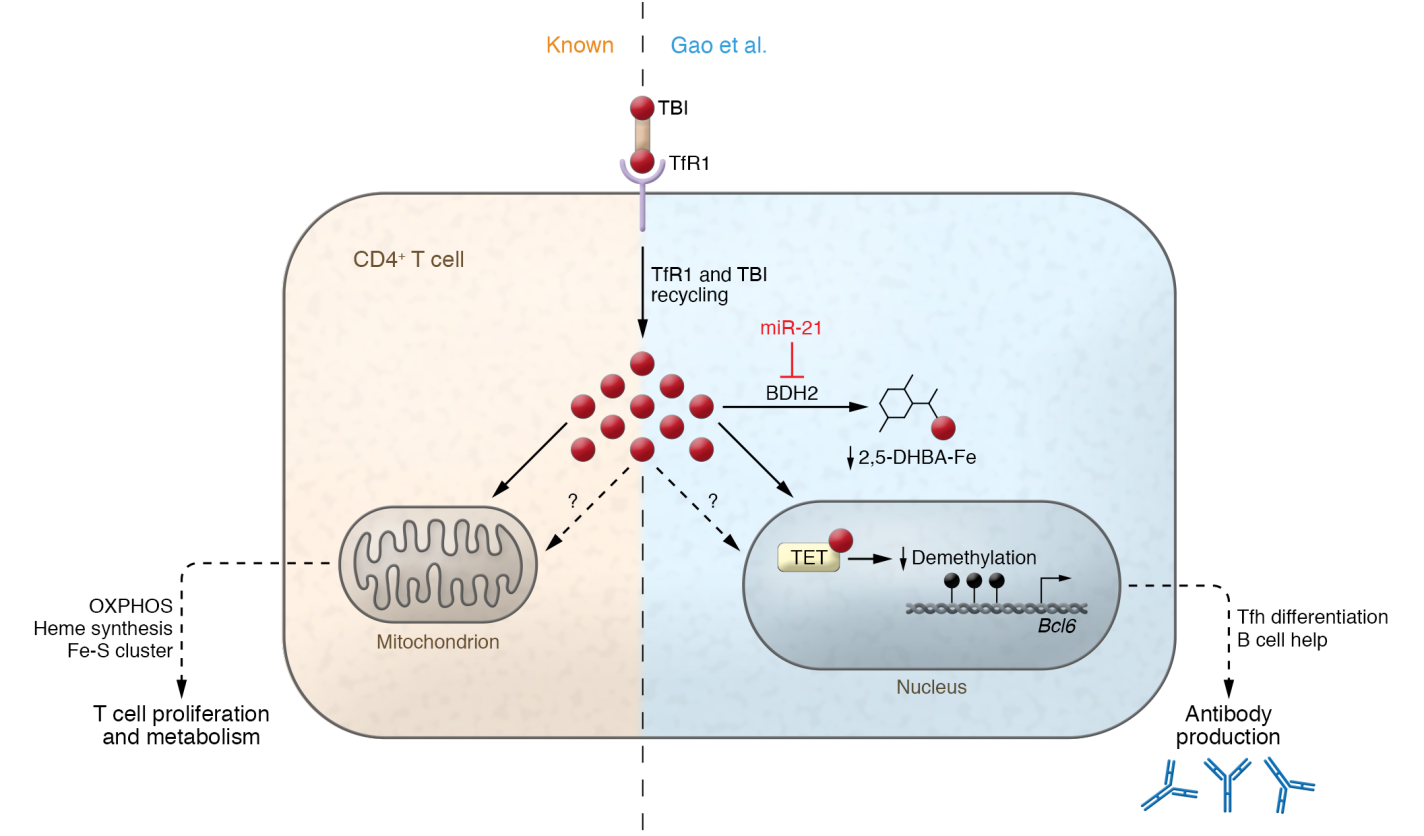
The miR-21/BDH2/Fe axis promotes DNA hydroxymethylation of the *BCL6* gene by regulating intracellular iron. Activated CD4^+^ T cells rapidly upregulate iron import machinery, increasing transferrin receptor 1 (TfR1) to support enhanced proliferative and metabolic requirements. Gao et al. (8) identified an additional role for iron in lupus CD4^+^ T cell differentiation and effector function. In CD4^+^ T cells from lupus models, miR-21 inhibited BDH2, the hydrogenase required for the synthesis of 2,5-DHBA, a cytoplasmic iron-binding molecule that safely assimilates excess iron. Loss of 2,5-DHBA increased the availability of Fe^2+^ in activated CD4^+^ T cells. Excess iron bound to and increased the activity of TET enzyme. This led to reduced DNA demethylation and enhanced gene expression of *BCL6*, which encodes the Tfh cell lineage defining transcription factor. Free iron may also influence parallel pathways. Notably, increased Tfh cells worsen lupus outcomes by helping B cells make more autoantibodies. TBI, transferrin-bound iron.

Gao et al. showed miR-21 to be overexpressed in lupus T cells, which correlated with Tfh cell frequency and disease activity (8). Furthermore, the authors demonstrated that the downregulation of BDH2 by miR-21 activated Fe^2+^-dependent TET enzymes that drove DNA hydroxymethylation and demethylation, promoting transcription of genes, including *Bcl6*, the master regulator of Tfh gene expression, and *Cd40l*, a key effector gene for Tfh cells (8). DNA demethylation is a mechanism known to activate CD4^+^ T cells in SLE and increase *CD40L* expression (12). However, *BCL6* expression has not been investigated in these studies. Gao et al. proposed a model whereby the miR-21/BDH2/Fe axis promotes DNA hydroxymethylation of the *BCL6* gene by regulating intracellular iron, which leads to increased differentiation of Tfh cells (ref. 8 and Figure 1). This mechanism applies to Tfh cells induced by either immunization with foreign antigens or autoimmune activation. This model is consistent with previous findings showing that targeting miR-21 is therapeutic in lupus-prone mice (13, 14).

## Translational importance

This study suggests that manipulating intracellular iron through systemic availability modulates humoral responses and ameliorates lupus by controlling the expansion of the Tfh cell population. Iron chelation may be therapeutic in diseases mediated by pathogenic autoantibodies, whereas iron supplementation may boost the production of protective antibodies against pathogens. In the context of autoimmune disease, reducing intracellular labile iron by administration of the hormone hepcidin has been shown to attenuate murine lupus nephritis (15).

The work presented in Gao et al. (8) raises a number of mechanistic considerations. Several studies have reported increased expression of miR-21 in SLE in response to the activation of the TLR7/type I IFN pathway, a major driver of lupus pathogenesis (16). It has also been postulated that hypoxia is the primary inducer of miR-21 (17). On the other hand, HIF-1α, the master regulator of the hypoxia response, suppresses Tfh cell differentiation in mice following lymphocytic choriomeningitis virus infection by inhibiting mTOR activation (18), suggesting that the relationship of the miR-21/BDH2/Fe^2+^ pathway with HIF-1 and mTOR activation should be explored. In addition, iron deficiency has previously been shown to diminish B cell responses by impairing cyclin E1 expression through the inhibition of H3K9me3/2 demethylation (19). Thus, the characterization of the miR-21/BDH2/Fe^2+^ axis in B cells is of interest, because *Bcl6* induces B cell differentiation in the germinal centers.

Other questions remain to be answered: How much does the transferrin receptor 1–mediated iron import machinery contribute to TET enzyme activation? What is the contribution of miR-21–induced labile iron accumulation toward cell proliferation or metabolism? Is iron shunted to meet cell differentiation requirements after fulfilling obligatory biological requirements, such as proliferation, metabolism, DNA synthesis? Answers to these inquiries could provide deeper insight into the importance of shuttling and delivery of iron into different cellular compartments in normal physiology and in disease.

The collective studies of Gao et al. (8) and others highlighted in this Commentary establish iron metabolism as a potentially druggable target in the pathogenesis of SLE that could synergize with existing immunosuppressive therapies. Consequently, mechanisms of cellular, as opposed to systemic, iron homeostasis in SLE warrant further study.

## Version 1 05/02/2022

Electronic publication
